# Cutting Out Stigma: Findings From a Pilot Implementation Trial of a Barbershop‐Based HIV Stigma Reduction and Health Promotion Intervention in the Southern United States

**DOI:** 10.1002/jia2.70195

**Published:** 2026-10-08

**Authors:** Jessica M. Perkins, Ruitian Xeno Hu, Madison Humphrey, Shawnika J. Hull, Konor von Kraut, Nathaniel King, Steven Nelson, Carolyn M. Audet, Zhihong Xeno Yu, Bryan E. Shepherd, Sharon Hurt, Aima A. Ahonkhai

**Affiliations:** ^1^ Peabody College Vanderbilt University Nashville Tennessee USA; ^2^ Medical Practice Evaluation Center Massachusetts General Hospital Boston Massachusetts USA; ^3^ Rutgers University, School of Information and Communication Rutgers New Jersey USA; ^4^ Luxury Mobile Barbering Nashville Tennessee USA; ^5^ Distinguished Gentlemen Barbershop Nashville Tennessee USA; ^6^ Vanderbilt University Medical Center Nashville Tennessee USA; ^7^ Street Works Nashville Tennessee USA; ^8^ Division of Infectious Diseases Massachusetts General Hospital Boston Massachusetts USA; ^9^ Center for AIDS Research Harvard University Boston Massachusetts USA

**Keywords:** barbershops, Black men, HIV, multimedia, prevention, stigma

## Abstract

**Introduction:**

Black Americans and individuals in the Southern United States bear a disproportionate burden of HIV. To address stigma, a key contributor to these inequities, we developed *Cutting Out Stigma* (*COS*), a barbershop‐based education and multimedia intervention.

**Methods:**

We recruited and trained Tennessee (U.S.) barbers as *COS* Men's Health Ambassadors and invited them to participate in a 6‐month *COS* pilot implementation trial from September 2024 to March 2025. *COS* barbers provided sexual health education, referrals to HIV (and other ancillary) services for barbershop clients and shared relevant study and barber‐created social media posts. Using the RE‐AIM framework, we assessed reach (social media and barbershop‐based), preliminary effectiveness (barber‐level HIV knowledge improvement, role effectiveness and resource connection), intervention adoption and implementation (feasibility, acceptability, appropriateness at baseline and endline). Data sources included validated pre‐ and post‐implementation surveys, social media analytics, weekly self‐report forms and in‐depth interviews (IDIs). Quantitative data were summarized descriptively, with mixed‐effects models estimating change over time; IDI analyses helped interpret effectiveness outcomes.

**Results:**

Sixty‐seven barbers completed COS training. Among 57 successfully contacted post‐training, 50 barbers (87.7%) enrolled in the pilot trial across 26 barbershops. The pilot reached an estimated 4464 barbershop client encounters, with 62.0% of barbers engaging ≥6 clients weekly. Estimated social media reach averaged 57,462 users monthly. Implementation outcomes remained high: baseline/endline scores were 4.8/4.8 for feasibility, 4.9/4.9 for acceptability and 4.8/4.9 for appropriateness (each out of 5). HIV knowledge improved following training, and barbers reported high effectiveness as health ambassadors (median effectiveness 8 of 10; IQR: 7–9). Barbers facilitated an estimated 3096 referrals, primarily for HIV testing (71.0%) and pre‐exposure prophylaxis (PrEP) (70.0%). IDIs helped explain these patterns: barbers described moving from transmission myths towards actionable understanding of HIV prevention, reported shifts in personal attitudes as they grew in their new role, and perceived normalizing HIV discussion and making resource referrals as “shop talk” that could reduce community stigma. Barbers also described client‐level early prevention behaviours (e.g. condom uptake, PrEP inquiries), though they felt fear of judgement created intention−behaviour gaps for a few clients.

**Conclusions:**

*COS* demonstrated high feasibility, acceptability and suggested preliminary effectiveness in reducing HIV stigma and promoting prevention through trusted barbershop settings. Integrating community‐based and digital strategies offers a promising model to tackle HIV stigma and advance Ending the HIV Epidemic goals.

## 1 Introduction

While significant advances in HIV prevention and treatment have been achieved over the past few decades, geographic and racial inequities have persisted as major obstacles to ending the HIV epidemic [1]. In 2022, more than half of all new HIV acquisitions in the United States occurred in the South, and the Black community has borne the brunt of the epidemic [2]. Black individuals comprise 12.0% of the U.S. population but account for 39.0% of new HIV diagnoses [3]. Black males face the highest lifetime risk of HIV (1 in 22), the highest age‐adjusted HIV‐mortality rates, and the greatest unmet need for prevention with pre‐exposure prophylaxis (PrEP) [4, 5]. Despite this burden, traditional health systems have struggled to engage those most in need. Deeply entrenched community stigma about HIV erodes each step of the HIV prevention and care continua, including testing, linkage to PrEP, linkage to care and viral suppression [6]. Accordingly, the National Institutes of Health (NIH) has identified stigma as a priority focus for research efforts, and elimination of HIV stigma as an important public health goal [7]. Nonetheless, few stigma interventions have been developed for and with Black men in the Southern United States [8]. Community‐based strategies leveraging trusted, culturally resonant spaces represent an important pathway to reducing HIV stigma, combating inequities and advancing Ending the HIV Epidemic (EHE) goals, a U.S. initiative to reduce new HIV acquisitions by 90% by 2030 through expanded testing, treatment, prevention and rapid response [9].

Barbershops, long recognized as trusted cornerstones of community life within Black communities, have served as effective venues for delivering health education and screening programmes for chronic diseases [10]. Barbers are viewed as confidants, and their shops as safe, familiar spaces where men gather regularly (often several times monthly) to converse, exchange advice and build community in addition to getting haircuts [10, 11, 12]. Though the feasibility of barbershop interventions for Black men in the United States has been described, most published interventions in Black barbershops have addressed cancer (50.0%) or hypertension (35.7%) [10, 13]. Only two interventions, *Fade Out HIV* and the *Barbershop Talk with Brothers Trial*, have specifically addressed sexually transmitted infection risk reduction for Black men through barbershops in the United States [13, 14]. Further, most barbershop‐based health interventions have not engaged barbers as implementers, thus missing critical opportunities to promote capacity building, community empowerment and sustainability [15].

In addition to community‐based interventions, multimedia interventions (spanning print media, television, radio initiatives and social media) have increased HIV awareness, promoted prevention‐focused health behaviours and increased HIV testing among key populations [16, 17, 18]. These approaches have been adapted for marginalized and minoritized communities both in the United States and globally [19, 20, 21, 22]. Platforms (like Facebook, TikTok and Instagram) are pivotal conduits for health promotion, offering tremendous capacity for information dissemination, communication enhancement and population reach [23]. Some mass media initiatives focusing on HIV education have indirectly reduced perceived HIV and related stigmas in the community; these have been more effective in high HIV prevalence settings, especially in low‐ and middle‐income countries [24, 25]. Social media interventions aimed at improving HIV prevention and treatment outcomes have been designed specifically to navigate the fear of stigma enacted against people living with HIV (PLWH), but not to combat community stigma [16, 19]. Few multimedia campaigns have aimed to reduce community HIV stigma, especially in the United States [26, 27, 28].

Integrating trusted community spaces, Black barbershops, with multimedia outreach offers a pathway to reduce community‐level HIV stigma and advance EHE goals. To address stigma as a key driver of HIV inequities in Black communities across the Southern United States, our team developed and implemented a multifaceted multimedia and barbershop‐based intervention—Cutting Out Stigma (*COS*). *COS* trains barbers as Men's Health Ambassadors to initiate and sustain positively framed conversations about HIV and sexual health with clients, while promoting awareness and facilitating referral pathways to HIV and community resources through in‐shop materials and coordinated social media content. The objective of this pilot implementation trial was to assess key RE‐AIM dimensions, including intervention reach, adoption and implementation outcomes (feasibility, acceptability and appropriateness), and to explore preliminary intervention effectiveness. While COS is designed to improve client‐level stigma and prevention outcomes, this pilot analysis focuses on upstream barber‐level effects including perceived effectiveness in COS role, changes in barbers’ HIV knowledge and referrals to community resources, as drivers of downstream client‐level impact.

## 2 Methods

### 2.1 Study Setting

The study is set in Tennessee's (TN) two highest‐burden counties, Shelby County (Memphis) and Davidson County (Nashville), which together account for 57.0% of the state's approximately 20,000 PLWH [29, 30]. Shelby County also has the highest HIV incidence among major U.S. metropolitan areas and is one of 48 EHE priority counties. Mirroring national trends, racial inequities exist across both counties; 80.9% of PLWH in Memphis and 51.3% of PLWH in Nashville are Black (compared to 51.0% and 25.0% of county residents, respectively) [31]. Statewide new HIV diagnoses and mortality rates among Black individuals are seven and six times those among White individuals, respectively [29, 30].

### 2.2 Intervention Description

Developed through a community−academic partnership, COS is a multifaceted barbershop‐based intervention that engages barbers as trusted community leaders and intervention co‐implementers [5, 17]. Grounded in Social Cognitive Theory, the Framework for Integrating Norms into Interventions for Stigma (FINIS) and the Health Belief Model, COS trains and supports barbers to provide HIV education, reduce HIV‐related stigma and facilitate linkage to HIV prevention, care, and community resources [32, 33, 34]. Together, these frameworks informed how the three‐component intervention utilizes interpersonal interactions and multimedia messaging to influence HIV‐related knowledge, attitudes, social norms, and engagement across the HIV prevention and treatment continuum:

1. First, barbers were *trained as Men's Health Ambassadors* through an intensive in‐person training focused on building knowledge and skills to support client engagement around HIV. Modules were delivered by subject matter experts and focused on the role of community health workers, men's sexual health, the prevention and HIV care continuum, stigma and its health impacts, communication strategies, mental health, implicit bias, faith‐based perspectives on HIV and lived experience with HIV. Local resources were also introduced through a community resource fair.
2. Second, interested barbers were consented and enrolled in the 6‐month pilot trial; they were encouraged to *initiate conversations* with clients about HIV and stigma in the context of sexual health and wellness during routine client interactions, and *to refer* their clients to HIV testing, PrEP services and other community resources (i.e. general and mental health services, legal resources, financial planning, etc.). To facilitate conversations, barbers were provided with a conversation starter guide and *COS*‐branded items (capes for barbers, smocks for clients, posters, etc.). To facilitate referral pathways, barbershops were stocked with PrEP brochures and community resource cards (with QR code links to local HIV/sexual health services and ancillary community resources) in addition to in‐shop resources *including* HIV self‐test kits and condoms.
3. Third, barbers implemented a *multimedia HIV education and stigma reduction campaign* using print, audiovisual and social media to help prompt barber−client conversations, and to provide HIV‐related education. Study‐provided in‐shop materials included HIV, PrEP, and community resource fact sheets and branded capes and smocks. A social media/marketing company with expertise in minority health campaigns helped the study team curate a social media calendar containing 26 posts introducing barbers as Men's Health Ambassadors, delivering HIV and wellness education, and supported barber‐generated videos, photos and text posts. Barbers and the study team posted on Instagram and Facebook according to a predeveloped calendar. Barbers were encouraged to post at prespecified times, use campaign hashtags and/or tag study accounts when possible [35]. Billboards were also displayed in each city (see Figure 1 for samples of multimedia materials).

**FIGURE 1:**
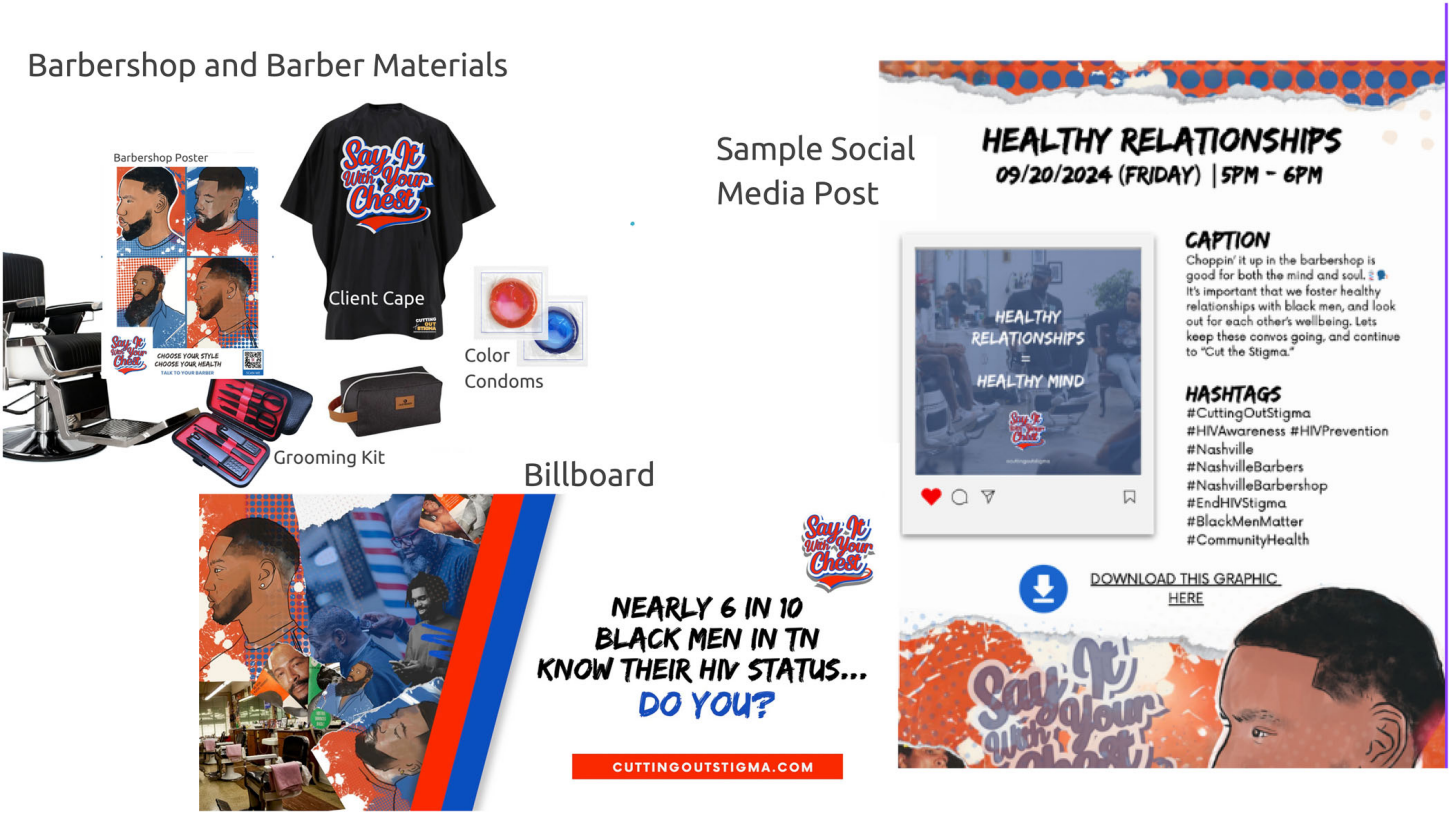
Representative examples of COS multimedia materials.

In addition to the initial training, during the trial, barbers participated in regular group coaching sessions with other barbers. Although the sessions were led primarily by study investigators, the groups functioned as supportive communities of practice focused on peer learning, troubleshooting and booster content to reinforce concepts from the initial in‐person training.

### 2.3 Recruitment, Enrolment and Remuneration

Barbers who worked in Shelby or Davidson County, were at least 18 years old, reported serving predominantly Black male clients and worked more than 20 h per week in their shop were recruited via: (1) our established academic−community partnership; (2) other community partners; and (3) local barber networks [36]. Eligible barbers were invited to complete the health ambassador training. Barbers who consented and completed the training were eligible for participation in the pilot trial.

The study was originally designed as a pilot trial for 10 barbers. However, strong interest from barbers and encouragement from community partners prompted us to expand participation. To maximize community engagement while remaining within available resources, we established intensive and standard participation groups. All barbers who consented and enrolled in the pilot trial were assigned to either an intensive data collection group or a standard data collection group. The intensive group included six barbers from three barbershops in Davidson County and four barbers from two barbershops in Shelby County (randomly sampled first by barbershop, then by trained barbers within the barbershop). The remaining participants were assigned to the standard data collection group (*n* = 40).

The barbers in the intensive group engaged in frequent reporting (via weekly self‐report forms), frequent interaction with the study team through weekly coaching calls and frequent social media posting (minimum once weekly), and provided endline in‐depth interviews (IDIs). Barbers in the standard group did not engage in weekly reporting nor endline interviews, had less frequent interaction with the study team (monthly coaching calls) and had a lesser requirement for social media posting (minimum once monthly). All barbers received a $500 stipend for participating in the Men's Health Ambassador training. Barbers in the intensive group received $800/month during the pilot trial, while all other barbers received $150/month for conducting study activities as described above.

### 2.4 Data Collection

Following Men's Health Ambassador training in summer 2024, participating barbers attended orientation meetings in September 2024. The trial was then conducted between 15 September 2024 and 15 March 2025. Guided by the RE‐AIM framework, we collected quantitative and operational data from participating barbers to assess reach, adoption, implementation and barber‐level indicators of preliminary effectiveness; maintenance was not assessed given the pilot design. HIV knowledge was assessed from all barbers participating in the pilot trial before and after Health Ambassador training. Other quantitative data were collected from all barbers at baseline (immediately after the Men's Health Ambassador training) and endline (after completion of the pilot trial) using validated instruments as described below. Operational data (weekly surveys) and qualitative data (IDIs) were collected from barbers in the intensive data collection group. Below, we describe how we defined and assessed each of these four RE‐AIM dimensions evaluated in this study.

*Intervention reach* refers to the absolute number, proportion and representativeness of individuals who participated in, received or were affected by the *COS* intervention. Barbershop‐based reach was quantified via operational data collected using self‐report forms completed weekly by barbers in the intensive group. Specifically, these forms elicited the number of clients who barbers engaged in conversations about focal topics and the number and types of service referrals provided to clients. Social media‐based reach is defined as the estimated number of unique users who view a post, and impressions are from all participating barbers using follower counts and engagement metrics (likes, comments, shares and other interactions) according to commonly used industry‐standard engagement formulas. Post‐level reach estimates were aggregated across participating barber accounts to generate overall estimates of social media exposure. Impressions were then estimated from reach using an average viewing frequency (1.5), reflecting standard digital marketing practice for accounting for repeated content exposure [37, 38].

Intervention *adoption* refers to the absolute number, proportion and representativeness of intervention agents who are willing to initiate the intervention. Because readiness and enthusiasm are theorized antecedents of adoption, we measured Organizational Readiness for Implementing Change (ORIC) and intervention enthusiasm to characterize barbers’ preparedness and motivation to engage in COS. While assessed from the perspective of individual barbers, ORIC captures perceptions of the broader implementation context and the capacity of the barbershop environment to support implementation. Enthusiasm was measured using a 5‐item instrument with responses measured on a 100‐point Visual Analogue Scale [39]. Readiness was measured using the validated 12‐item ORIC instrument with responses measured (on a 5‐point Likert scale) [40]. These were assessed immediately following health ambassador training and after intervention implementation. We then quantified adoption as the proportion of barbers who enrolled in the implementation trial after the training workshop.

Intervention *implementation outcomes* for this study refer to intervention acceptability (AIM), appropriateness (IAM) and feasibility (FIM). These outcomes were evaluated at baseline (after the Men's Health Ambassador training) and endline using three validated 4‐item scales [41].

Preliminary intervention *effectiveness* was assessed using measures expected to influence client‐level outcomes. These included changes in barber HIV and PrEP knowledge, perceived role effectiveness and community resource referral activity. These outcomes are viewed as upstream drivers of subsequent client‐level change. HIV knowledge was assessed before and after the health ambassador training for all barbers using a 21‐item knowledge questionnaire (KQ) combining items from two validated questionnaires (HIV‐KQ‐18 and HIV‐KQ‐45). Perceived effectiveness as a Men's Health Ambassador (during the previous week on a 10‐point scale) was quantified via self‐reported data collected on the weekly form from barbers in the intensive group. These weekly forms also elicited the number of times barbers referred clients to community resources.

Additionally, semi‐structured IDIs were conducted via Zoom. Consistent with an explanatory logic [42], the barber accounts could illuminate why the intervention was, or was not, perceived as effective. The IDI guide elicited barbers’ knowledge gained and personal experiences as *COS* ambassadors in addition to perceptions about client impact (attitudes, sexual health interest, behaviour). Integration of the quantitative and qualitative strands occurred principally at the level of interpretation [43]. Interviews lasted 45–60 min and were audio‐recorded, transcribed verbatim, and de‐identified.

### 2.5 Data Analysis

Descriptive statistics were used to summarize barber demographics, enthusiasm, readiness (ORIC), and acceptability, appropriateness and feasibility. *T*‐tests and chi‐squared tests (or Fisher's exact test when the number of any category was less than 5) were used to compare continuous and categorical variables, respectively. Barbershop‐based reach and referrals were estimated by extrapolating the number of client interactions reported by barbers participating in intensive data collection to the full cohort (using conservative estimates), providing an overall estimate of client encounters and number of referrals facilitated. We calculated average combined monthly social media‐based reach and impressions across individual and combined platforms (Instagram and Facebook). Paired *t*‐tests were used to compare change in HIV knowledge before and after the Men's Health Ambassador training. Mixed‐effects models were used to compare baseline versus endline enthusiasm, readiness and implementation outcomes. A mixed‐effects model was also used to assess change over time in reported self‐efficacy in the health ambassador role by *COS* barbers during the pilot intervention.

Interview data were analysed using rapid qualitative analysis [44, 45, 46, 47, 48] within a directed (deductive) content‐analytic approach [49]. The RE‐AIM effectiveness dimension provided the a priori analytic framework, with barbers’ HIV knowledge, role and referrals serving as predetermined domains, and stigma reduction examined as a cross‐cutting theme across all three. Rather than coding verbatim, analysts reviewed each transcript and summarized it directly into a structured template keyed to these domains, capturing exemplar quotes alongside each summary. Summaries were then charted into a matrix (participants × domains), following the matrix logic of the Framework Method [50], to enable cross‐case comparison. Within each domain, analysts identified recurring patterns and sub‐patterns within the predetermined domains, deductively derived rather than inductively emergent themes.

Trustworthiness was supported through independent template calibration and consensus between two analysts, verification of a subset of summaries against source transcripts and iterative team review of identified patterns. Quantitative effectiveness findings and the qualitative patterns were merged and interpreted together on a domain‐by‐domain basis [43]. For each effectiveness domain, the team characterized how qualitative accounts corroborated the quantitative results, elaborated any mechanisms potentially behind the results and any divergence from the quantitative results. The team also summarized barber‐reported early client indicators and theorized downstream impacts. Artificial Intelligence assistance was used to review the finalized summaries, quotes and joint display, assisting the team in honing the wording of pattern descriptions; all analytic decisions remained with the study team.

### 2.6 Ethics

The study protocol was reviewed and approved by the Vanderbilt University Medical Center Institutional Review Board (IRB #240024). Informed consent was obtained from all participating barbers prior to enrolment in the study and participation in study activities.

## 3 Results

A total of 79 barbers were recruited and consented for participation in the Men's Health Ambassador training; 69 barbers attended and completed the training. Fifty‐seven of sixty‐seven barbers were then successfully contacted by the study team and recruited into the pilot trial, and 50 barbers enrolled in the trial (Figure 2). Missing endline data were primarily attributable to loss to follow‐up and scheduling constraints during the endline data‐collection window rather than to study withdrawal. The 50 barbers in the *COS* pilot trial were equally distributed across Davidson (*n* = 25) and Shelby (*n* = 25) counties and represented 26 barbershops. Most participants identified as male (94.0%, *n* = 47) and as Black/African American (96.0%, *n* = 48). Sixty‐eight percent (*n* = 34) reported greater than high school education. More than half (54.0%, *n* = 27) had over 10 years of professional experience, and 22.0% (*n* = 11) were also barbershop owners. Barbers in the intensive group were older than those in the standard group (40.3 SD 10.7 vs. 33.9 SD 8.9, *p* = 0.07) but were similar for other demographic characteristics (Table 1).

**FIGURE 2:**
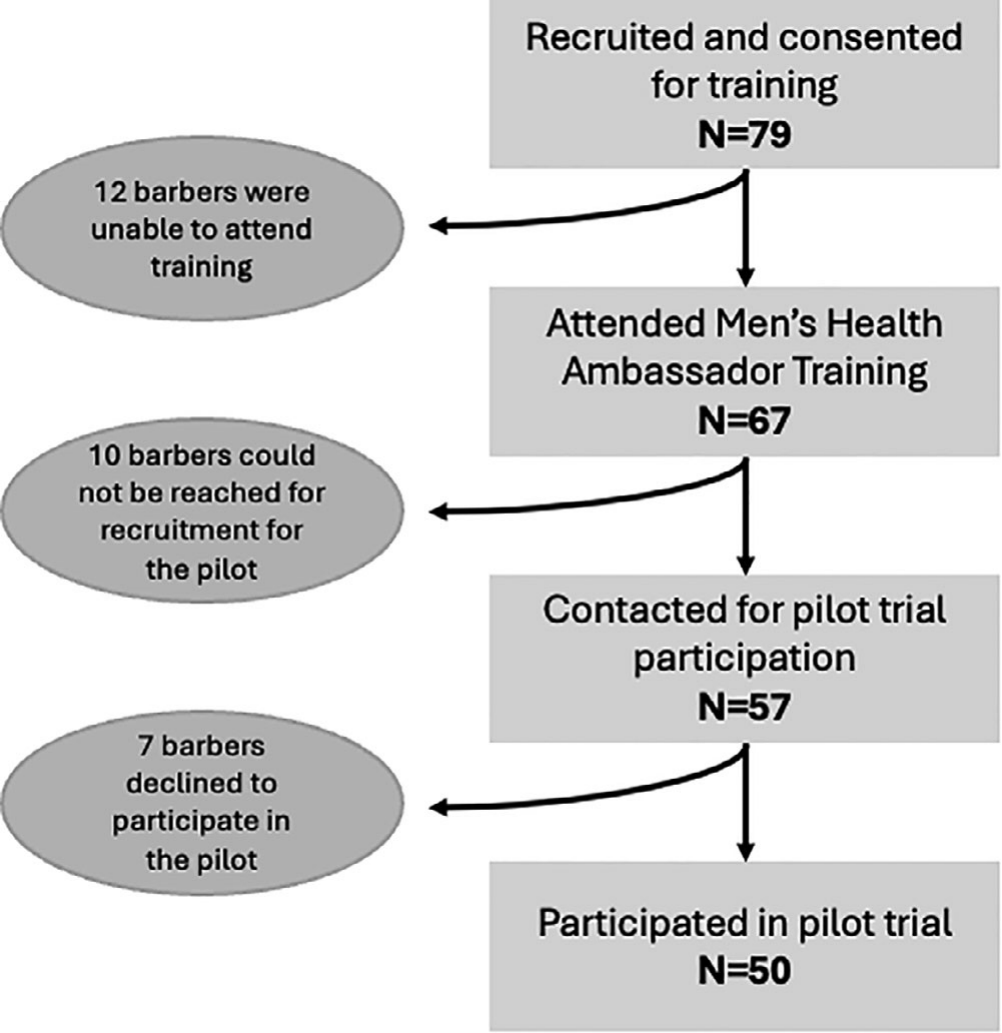
CONSORT flow diagram of *Cutting Out Stigma (COS)* intervention (barber) implementers.

**TABLE 1:** Baseline characteristics of barbers participating in *Cutting Out Stigma* implementation trial.

Demographic characteristics	Overall (*n* = 50)	Standard group (*n* = 40)	Intensive group (*n* = 10)	*p*‐value
Mean age in years (SD)	39.0 (10.6)	40.3 (10.7)	33.9 (8.9)	0.07
Biological sex				0.50
Male	47 (94.0%)	38 (95.0%)	9 (90.0%)	
Female	3 (6.0%)	2 (5.0%)	1 (10.0%)	
Self‐reported race/ethnicity				1.00
Black/African‐American	48 (96.0%)	38 (95.0%)	10 (100.0%)	
Hispanic/Latinx	1 (2.0%)	1 (2.5%)	0 (0.0%)	
Other	1 (2.0%)	1 (2.5%)	0 (0.0%)	
Level of education				0.17
High school or less	16 (32.0%)	15 (37.5%)	1 (10.0%)	
Some college	16 (32.0%)	10 (25.0%)	6 (60.0%)	
Technical school	9 (18.0%)	7 (17.5%)	2 (20.0%)	
Associate's/Bachelor's degree or higher	9 (18.0%)	8 (20.0%)	1 (10.0%)	
Years of barbering experience				0.48
<1 year	4 (8.0%)	3 (7.5%)	1 (10.0%)	
1–10 years	19 (38.0%)	14 (35.0%)	5 (50.0%)	
>10 years	27 (54.0%)	23 (57.5%)	4 (40.0%)	
Barbershop owner				0.42
Yes	11 (22.0%)	10 (25.0%)	1 (10.0%)	
No	39 (78.0%)	30 (75.0%)	9 (90.0%)	
Barbershop location				0.72
Shelby County	25 (50.0%)	21 (52.5%)	4 (40.0%)	
Davidson County	25 (50.0%)	19 (47.5%)	6 (60.0%)	
Prior HIV‐related conversation, behaviours and social networks
Talked with anyone outside the barbershop about HIV‐related resources, prevention and/or treatment in the past 6 months
Yes	21 (42.0%)	19 (47.5%)	2 (20.0%)	0.16
No	29 (58.0%)	21 (52.5%)	8 (80.0%)	
Tested for HIV in the past 6 months
Yes	20 (40.0%)	15 (37.5%)	5 (50.0%)	0.49
No	30 (60.0%)	25 (62.5%)	5 (50.0%)	
Sought out information about PrEP in the past 6 months
Yes	2 (4.1%)	2 (5.1%)	0 (0.0%)	1.00
No	47 (95.9%)	37 (94.9%)	10 (100.0%)	
Personally know anyone (friend or family) on PrEP
Yes	6 (12.0%)	5 (12.5%)	1 (10.0%)	1.00
No	44 (88.0%)	35 (87.5%)	9 (90.0%)	

*Note*: *p*‐values derived from Fisher's exact test for categorical variables and independent samples *t*‐test for continuous variables.

Abbreviations: PrEP, pre‐exposure prophylaxis; SD, standard deviation.

### 3.1 Reach

Sixty‐two percent of barbers reported engaging in conversations with an average of six or more clients per week. Applying this proportion to the full cohort of 50 participating barbers and conservatively assuming six client encounters per week over the 24‐week intervention period yielded an estimated cumulative reach of approximately 4464 client encounters. The average number of followers per individual social media account across all barbers was 1025, with an estimated monthly reach of 44,609.8 (Instagram) and 9585.7 (Facebook). In addition, the *COS* study's social media accounts accumulated 140 followers and achieved an estimated average monthly reach of 1785.8 (Instagram) and 1480.5 (Facebook) (Table 2).

**TABLE 2:** *Cutting Out Stigma* implementation trial outcomes in the RE‐AIM (Reach, Effectiveness, Adoption, Implementation, Maintenance) framework.

Implementation outcome and measure	Measure (%) or *N*	*p*‐value
**Reach**		
Distribution of self‐reported average number of clients with whom barbers discussed focal topics weekly		
0–5 clients	38.0%	
6–10 clients	39.0%	
11–15 clients	13.0%	
16–20 clients	6.0%	
20+ clients	4.0%	
Average number of followers to the *COS* social media account	140.0	
Average number of followers to the social media accounts of barbers in *COS*	1025.0	
Average Estimated Monthly COS social media reach (IG)	1785.8	
Average Estimated Monthly COS social media impressions (IG)	2165.3	
Average Estimated Monthly COS social media reach (FB)	1480.5	
Average Estimated Monthly COS social media impressions (FB)	2645.6	
Average Estimated Monthly barber social media reach (IG)	44,609.8	
Average Estimated Monthly barber social media impressions (IG)	66,915.0	
Average Estimated Monthly barber social media reach (FB)	9585.7	
Average Estimated Monthly barber social media impressions (FB)	14,378.6	
Average Estimated Total Monthly social media reach (Study + Barbers) across platforms	57,461.8	
Average Estimated Total Monthly social media impressions (Study + Barbers) across platforms	86,104.5	
**Effectiveness**		
Distribution of self‐reported average number of weekly referrals to services		
0–5 referrals	57.0%	
6–10 referrals	25.0%	
11–15 referrals	8.0%	
16–20 referrals	4.0%	
20+ referrals	6.0%	
Distribution of services referred to weekly		
HIV testing	71.0%	
Pre‐exposure prophylaxis (PrEP)	70.0%	
HIV service organization	31.0%	
Primary care physician	49.0%	
Health insurance	34.0%	
Legal service	13.0%	
Other	11.0%	
Self‐reported effectiveness as Men's Health Ambassador (median, IQR)	8 (7−9)	
HIV knowledge gains (scale 0–21)	Mean (SD, *n*)	<0.01
Before training	15.0 (3.4, *n* = 50)	
After training	16.9 (3.3, *n* = 50)	
**Adoption**		
Acceptance rate for intervention participation	*n* = 50 of 57 (87.7%)	
Davidson County	*n* = 25 of 31 (80.6%)	
Shelby County	*n* = 25 of 26 (96.2%)	
Enthusiasm (scale 0–100)	Mean (SD, *n*)	0.12
Baseline	94.01 (13.6, *n* = 50)	
Endline	89.93 (16.5, *n* = 36)	
Readiness (scale 1–5)		0.43
Baseline	4.64 (0.5, *n* = 50)	
Endline	4.75 (0.3, *n* = 36)	
**Implementation**		
Feasibility (scale 1–5)		0.85
Baseline	4.80 (0.4, *n* = 50)	
Endline	4.84 (0.3, *n* = 36)	
Acceptability (scale 1–5)		0.47
Baseline	4.88 (0.3, *n* = 50)	
Endline	4.94 (0.1, *n* = 36)	
Appropriateness (scale 1–5)		0.40
Baseline	4.79 (0.5, *n* = 50)	
Endline	4.88 (0.3, *n* = 36)	

*Note*: HIV knowledge *p*‐value derived from paired *t*‐test comparing scores before and after Men's Health Ambassador training. Baseline versus endline *p*‐values for implementation outcomes derived from mixed‐effects models. Fifty barbers participated in the pilot trial and completed baseline data collection, and 36 barbers completed study end data collection.

Abbreviations: COS, Cutting Out Stigma; FB, Facebook; IG, Instagram; IQR, interquartile range; PrEP, pre‐exposure prophylaxis.

### 3.2 Adoption

Among barbers who participated in the Men's Health Ambassador training and were successfully contacted after the training (*n* = 57), intervention adoption was 87.7% (*n* = 50) overall (80.6% [*n* = 25 of 31] in Davidson County and 96.2% [*n* = 25 of 26] in Shelby County). Among all barbers participating in the pilot intervention, endline measurements were obtained for 36 of the 50 barbers (72.0%). Enthusiasm and readiness scores remained consistently high and similar between baseline and endline. Mean enthusiasm assessed on a 0–100 point scale was 94.0 at baseline (*n* = 50) versus 89.9 at endline (*n* = 36) (*p* = 0.12), and mean readiness assessed on a 1–5 scale was 4.64 at baseline (*n* = 50) versus 4.75 at endline (*n* = 36) (*p* = 0.43).

### 3.3 Implementation

Implementation outcome scores remained consistently high across both timepoints, with no statistical differences observed between baseline and endline: feasibility score (4.80 at baseline [*n* = 50] vs. 4.84 at endline [*n* = 36]; *p* = 0.85); acceptability (4.88 at baseline [*n* = 50] vs. 4.94 at endline [*n* = 36]; *p* = 0.47); and appropriateness (4.79 at baseline [*n* = 50] vs. 4.88 at endline [*n* = 39]; *p* = 0.40).

### 3.4 Effectiveness

Barbers’ HIV knowledge increased significantly following the Men's Health Ambassador training (mean score of 14.7 before the training vs. 16.8 after the training, *n* = 67, *p*< 0.001). Barbers in the intensive group rated their own effectiveness as ambassadors highly at baseline, where the median score was 8 of 10 (*n* = 10) with an IQR of 7–9. Effectiveness scores remained stable during the 6‐month pilot (*p* = 0.54). Forty‐three percent of barbers reported referring six or more clients each week to community resources. Applying this proportion to the full cohort of 50 participating barbers and conservatively assuming six referrals per week over the 24‐week intervention period yielded an estimated 3096 total referrals. Most referrals involved HIV testing (71.0%) and PrEP (70.0%), though barbers also referred clients to primary care (49.0%), health‐insurance resources (34.0%), and other social and legal services (13.0%).

Three patterns were identified within the predetermined effectiveness domains (around barber HIV knowledge, role effectiveness and community referrals), with stigma reduction as a cross‐cutting theme. First, barbers described shifting from viewing HIV as fatal or limited to certain groups to seeing it as a manageable, preventable condition affecting their communities. Initial training and ongoing coaching increased familiarity with PrEP and “undetectable is untransmittable” or U = U, reflected in a significant post‐training gain in HIV knowledge. Barbers felt this knowledge equipped them with ways to initiate conversations, address myths and guide clients accurately. They described how their certified expertise and consistent, accurate information built credibility with clients and trust in them as community leaders.

Second, barbers described moving from a sense of blame to openness, both in themselves and in their clients. Several related how increased knowledge and contact with PLWH (through *COS* training or personal relationships) reduced their own HIV‐related fear and judgement while fostering empathy and openness to clients from all backgrounds. Half reported experiencing HIV‐related stigma by association, but over time developed confidence in addressing these assumptions and using such interactions to normalize HIV conversations and challenge misconceptions. Although a few noted privacy constraining some conversations, most reported growing comfort discussing sexual health, with HIV talk becoming normalized as “shop talk.” Some reported less stigmatizing language in their shops, by clients and barbers alike. Barbers felt these changes, reinforced by practice having difficult conversations and by COS's positive‐framed social and visual cues in their shop and social media environments, helped them fulfil and grow in their role as Men's Health Ambassadors and trusted community leaders, contributing to community stigma reduction. Consistent with these accounts, barbers rated their own effectiveness in this role highly. Many barbers also reported discussing HIV beyond the barbershop, with a few asked by parents to discuss prevention with their children.

Third, some barbers reported these conversations translating into referrals and early client prevention engagement; they observed greater condom uptake, requests for testing information and inquiries about accessing PrEP. These sometimes led to referrals to community‐based resources for additional support. Quantitatively, almost half of barbers reported making more than five referrals each week, most often for HIV testing and PrEP. Some modelled testing themselves, signalling comfort and confidence with prevention while reducing stigma. Barbers emphasized that repeated, stigma‐free dialogue and visual reminders of COS, community resources and prevention kits helped clients move from misinformation to awareness to prevention. A few also described persistent intention−behaviour gaps driven by fear of judgement and lack of privacy, gender norms shaping what men discuss and do publicly, and client‐perceived invincibility. Table 3 provides exemplar quotes for each pattern, and Table S1 provides a joint display integrating qualitative and quantitative results.

**TABLE 3:** Evidence of preliminary intervention effectiveness and exemplary quotes from *Cutting Out Stigma's* (*COS*).

Patterns	Sub‐patterns	Exemplar quotes
**From myths to actionable understanding**	HIV reframed from fatal and exceptional to manageable and preventable	*“I thought it was a terminal death sentence… learning that there's a pill you can take regularly… you can live a good, full, healthy, awesome life.”* [Barber A] *“I probably was clueless… never heard of PrEP.”* [Barber B] *“We were like babies… a lot of people thought you had to be gay to get… I don't have that belief anymore.”* [Barber F]
	Trusted shop talk drives curiosity (especially around PrEP) and knowledge diffusion (especially among youth)	*“Our conversations became robust…I was a trusted source of information.”* [Barber A] *“People… would see me in‐person… ‘What is [COS]?’… great opportunity for me…to explain.”* [Barber D] “The main hype was about…that PrEP pill.” [Barber C] *“We had younger kids talking about [PrEP]… parents would let me talk to them about it.”* [Barber C] “*We definitely changed their opinions and helped them realize, you know, this is something serious. It's not something to joke about.”* [Barber B]
**From blame to openness and community leadership**	Humanizing contact and non‐judgement reduce fear, blame and derogatory talk	*“Hearing [PLWH] testimonies… it changed my perspective.”* [Barber G] *“Not judging… helped [clients] open up… cutting out the stigma… made it better.”* [Barber A] *“Shook their hand…gave him a hug… enlightening…great to see somebody [PLWH] thriving.”* [Barber E]
	Normalizing HIV talk increases comfort, but privacy and gender norms still constrain openness	“*Being able to have these conversations… brought me closer to them… makes [clients] feel like somebody cares.”* [Barber A] *“Stigma… still there, but it's lightened up… more comfortable conversation to have now than it was six months ago.”* [Barber E] *“I learned it didn't make them feel uncomfortable, you know, because they would come back.” [Barber B]* *“Gay clients… didn't want to open up… because of the stigma.”* [Barber E] “*Probably a little bit of stigma, the way that PrEP was advertised…at first…always…homesexual couples.”* [Barber I]
**Translating conversation into referrals and action**	Talk‐to‐action beginnings: condom uptake, testing modelling and PrEP inquiries	*“We had the condoms… some of the young guys grab a handful.”* [Barber C] *“Client …was willing to go to a local clinic with me… asking questions and being interested in PrEP.”* [Barber H] *“I… get tested quarterly… no reason you[client] can't… once or twice a year.”* [Barber A]
	Intention–behaviour gaps persist due to anticipated stigma and entrenched norms	*“Can't take [self‐test] at home… [client said] my old lady gonna think I've been doing something… if somebody saw you take it, they'd be like, oh he might think he has AIDS.’”* [Barber B] *“They did go… I don't think they actually got the prescription…but them being option [to go]…that's the first start.”* [Barber H] *“By the end… some started to care. Some continued not to.”* [Barber F]

*Note*: Quotes drawn from interviews with barbers in the intensive data collection arm (*n* = 10).

Abbreviations: PLWH, people living with HIV; PrEP, pre‐exposure prophylaxis.

## 4 Discussion

This pilot implementation trial provides early evidence that *COS*, a multicomponent barbershop‐based and multimedia intervention, can be feasibly implemented in the Southern United States. COS uses trusted barbers to shift norms about HIV in the context of men's sexual health and wellbeing. This approach holds promise for reducing community HIV stigma among Black men by transforming everyday social spaces into hubs for dialogue and connection to care. *COS* demonstrated consistently strong performance across implementation domains, extensive reach across both barbershop‐based and social media channels, and improvement in barber‐level outcomes that may support downstream reductions in community HIV stigma and improvements in HIV prevention engagement. Further, by leveraging a community‐engaged strategy that operates at both the interpersonal and community levels, *COS* extends the reach of traditional public‐health infrastructure to those most affected by inequities in HIV prevention and care.

*COS* demonstrated both high adoption and robust pre‐ and post‐implementation outcomes from the perspective of *COS* barbers. Nearly all barbers contacted after the Men's Health Ambassador training enrolled in the pilot, reflecting early buy‐in despite operating in communities where HIV stigma remains deeply entrenched [51, 52]. In our team's prior formative work, one TN barber captured this reality, remarking, “*I'd rather get shot than say I got AIDS*” [53]. Against this backdrop, the high adoption of *COS* underscores the intervention's resonance and credibility among Black barbers. High adoption may have been driven, at least in part, by barbers’ enthusiasm about the intervention, and their perceived readiness to implement it—perceptions that remained stable across the 6‐month pilot. Barbers reported sustained feasibility, acceptability and appropriateness, suggesting that barbershop‐based health conversations, client referrals and social media engagement were readily integrated into barbers’ routines and aligned with goals of promoting health and reducing HIV stigma.

This study builds on growing evidence supporting the feasibility, acceptability and appropriateness of barbershop‐based and multimedia interventions as effective vehicles for health promotion among Black men. In one review of 13 barbershop‐based health promotion interventions, intervention adoption among barbershops ranged from 62.4% to 90.6% from the trials that reported on this outcome [54]. Another systematic review of health promotion interventions in U.S. Black barbershops (*n* = 10) and hair salons (*n* = 4) noted that feasibility outcomes, when reported, were favourable. However, outcomes were not always explicitly stated, nor were standardized measures always utilized [10]. Further, only about half of the interventions engaged barbers or stylists as implementers, and none explicitly focused on stigma.

Reach of *COS* was considerable, both within barbershops and across social media platforms. Nearly half of barbers reported referring one client daily on average to community resources, emphasizing the value of embedding health promotion within the broader context of social and structural needs. The scale of *COS*’s social media reach exceeded that of many other social media interventions, possibly reflecting amplification achieved through barbers’ own social media followings; their online presence extended engagement well beyond the physical shop and the social media reach well beyond that achieved by the study team [55, 56]. These findings contribute to the growing evidence supporting the feasibility and reach of multimedia and social media–based strategies across the HIV prevention and treatment continuum. In a recent systematic review of 14 social media–driven HIV interventions, half incorporated in‐person components, underscoring the added value of hybrid models that integrate interpersonal and digital engagement [55, 56, 57]. However, most such interventions have primarily focused on men who have sex with men (MSM), and few have been designed to engage Black heterosexual men or those who may not identify with MSM‐focused messaging. This distinction is critical as intersecting stigma related to HIV, race and sexuality remains pervasive [58, 59]. In TN, where *COS* was piloted, 46.0% of Black PLWH reported transmission risk factors other than male‐to‐male sex, underscoring the need for approaches that resonate across the full spectrum of Black men's lived experiences [31].

Findings suggest that *COS* was associated with shifts in upstream barber‐level knowledge, comfort with positively framed, anti‐stigma HIV‐related conversation, and perceptions of personal and community HIV stigma, and barber‐observed early downstream prevention behaviours among clients consistent with effective published stigma‐reduction strategies [60]. Barbers reported greater understanding of HIV transmission and prevention, increased comfort discussing sexual health and observed early signs of behaviour change among clients—collectively indicating progress along the cognitive, affective and behavioural pathways essential to stigma reduction [34]. Barbers emphasized that connection to PLWH, whether through *COS* training or personal experiences, was transformative in reducing barbers’ fear and moral judgement, which then indirectly changed how they spoke about HIV with their clients. Barbers also described barriers rooted in the need for privacy, anticipated stigma by association (with some clients initially interpreting barbers’ willingness to discuss HIV as evidence of personal HIV status), gender and sexuality norms, and client‐perceived invincibility, underscoring the need for stigma‐reduction interventions that are nuanced, responsive to intersecting forms of stigma and that equip ambassadors to navigate sensitive discussions. Group‐based discussion on these barriers and associated role‐play during the ongoing coaching helped barbers think through some of these challenges over time, and barbers increasingly used these interactions to normalize HIV conversations and dispel misconceptions.

The three effectiveness domains examined here (barber HIV knowledge, role effectiveness and referrals) map onto the cognitive, affective and behavioural components through which HIV stigma is theorized to form and be reduced [34]. Gains in barbers’ HIV knowledge correspond to the cognitive component, dismantling the fatal‐disease myths and misconceptions that sustain stigma; barbers’ reduced fear and moral judgement, increased comfort discussing sexual health and growth in the trusted Men's Health Ambassador role correspond to the affective component; and the translation of shop conversations into referrals, testing modelling and barber‐observed early client prevention behaviours corresponds to the behavioural component. Stigma reduction was not confined to any one domain but operated across all three, with accurate knowledge supporting less fearful attitudes that in turn fostered more open, stigma‐free dialogue and action. This cross‐cutting pattern is consistent with theoretical models holding that durable stigma change requires simultaneous shifts in what people know, how they feel and how they act.

That this stigma reduction unfolded through barbers acting as credible, trusted peers reflects a central tenet of stigma theory: repeated, non‐judgemental interactions with credible community members transform the social norms that perpetuate stigma [34, 61]. These patterns also align with evidence from meta‐analyses demonstrating that interventions characterized by repeated contact, interpersonal dialogue and multi‐component delivery produce larger and more sustained effects on HIV‐related stigma than single‐session or didactic approaches [62, 63]. *COS*’*s* combination of structured training, ongoing coaching and continued barbershop and social‐media engagement created multiple opportunities for reflection, reinforcement and normalization, conditions identified as critical for durable attitude and behaviour change [64]. Moreover, the hybrid barbershop‐based and social media dissemination model represents an evolution from prior interventions that primarily targeted individual knowledge or healthcare provider bias [55]. Taken together, the observed improvements in HIV knowledge, reductions in stigma‐related discomfort and early behavioural indicators among barbers and clients reinforce the potential of *COS* as a scalable model for community‐driven, multilevel stigma reduction in the U.S. South.

This non‐randomized pilot study has limitations, including potential reporting bias; reliance on barbers’ self‐reported data during a short follow‐up period limits generalizability and the ability to assess sustained impact. Barbershop‐based reach across all barbers was estimated from reports by barbers in the intensive group only and thus may over‐ or underestimate total barbershop‐based reach. Although client‐level outcomes are critical for evaluating intervention effectiveness, analyses of client HIV stigma and perceptions of COS are beyond the scope of this implementation‐focused manuscript and will be reported separately. In addition, future analyses should examine contextual barriers and facilitators to implementation using qualitative and operational data collected throughout the trial. Despite these limitations, this study has several strengths. *COS* is among the few stigma reduction interventions developed for and with Black men in the Southern United States, addressing a critical gap in HIV stigma research. Its community‐engaged design, integrating barbershop‐based and digital components, allowed *COS* to operate simultaneously at interpersonal and community levels. Spotlighting social media analytics alongside in‐shop engagement metrics enabled an extensive evaluation of both in‐person and digital reach, extending the evidence base for hybrid stigma‐reduction models. Further, we used a validated implementation framework and tools to guide assessment.

## 5 Conclusions

*COS* is a feasible, acceptable and promising approach for reducing HIV stigma among Black men in the Southern United States. By leveraging barbers as trusted community leaders and integrating both in‐shop and social media strategies, *COS* demonstrates how these strategies may work to address community HIV stigma while promoting HIV prevention, testing and care. A larger‐scale, hybrid implementation‐effectiveness trial of *COS* is warranted to rigorously assess its effects on community stigma, behavioural change and clinical outcomes, and to examine sustainability and scalability.

## Author Contributions

AAA conceptualized the study. S Hull, NK, SN, ZY, S Hurt and AAA developed the methodology. JMP, ZY and AAA performed data curation. JMP and AAA conducted the investigation. BES performed validation. JMP, RXH, MH and AAA wrote the original draft. AAA provided supervision, secured funding acquisition, administered the project and contributed resources along with S Hurt. All authors read and approved the final version of the manuscript for publication.

## Conflicts of Interest

The authors declare no conflicts of interest.

## Supporting information




**Supplementary Material**. In‐depth interview questions around effectiveness of barbers serving as Men's Health Ambassadors with *Cutting Out Stigma*.

**Table S1**. Integration of quantitative effectiveness results and qualitative findings from Cutting Out Stigma, with barber‐reported early client indicators.

## Data Availability

The data that support the findings of this study are available on request from the corresponding author. The data are not publicly available due to privacy or ethical restrictions.
