# Supplementary material for: Cutting Out Stigma: Findings From a Pilot Implementation Trial of a Barbershop‐Based HIV Stigma Reduction and Health Promotion Intervention in the Southern United States
Source: J Int AIDS Soc. 2026 Oct 8;29(Suppl 5):e70195. doi: 10.1002/jia2.70195 (PMC13650421; doi:10.1002/jia2.70195)
Supplement: Supplementary file 1 [file JIA2-29-e70195-s001.docx]

Supplemental Material. In-Depth Interview Questions around Effectiveness of Barbers Serving as Men’s Health Ambassadors with *Cutting out Stigma*.

1. What aspects of serving as a men’s health ambassador with *Cutting out Stigma* did you enjoy and why*?*

*Potential probes:*

a. *What was comfortable?*
b. *What aspects of your role were easy?*

2. What aspects of serving as a men’s health ambassador with *Cutting out Stigma* were challenging and why*?*

*Probes:*

a. *What was uncomfortable?*
b. *What aspects of your role did you dislike?*

3. How did your experience change over time?
4. Would you share a bit about your own thoughts and opinions (mindset) about HIV that you brought into this project and to what extent they changed over time?

*Probes:*

a. *What have you learned about your own biases during the program?*
b. *What about towards people with HIV?*

5. What about your clients’ biases about men’s sexual health and HIV in particular…what did you learn over the past 6 months?

*Probes:*

a. *How do your biases differ from your clients?*
b. *How did the biases change?*

6. How have your clients responded to your work as a men’s health ambassador?

*Probes:*

a. *What feedback/responses have you received about:*
   - *Conversations you have initiated?*
   - *Swag/merch/visuals in your shop?*
   - *Social media posts?*

7. How has being a men’s health ambassador affected your relationship with your clients?
   a. *How much have any clients or other barbers in your shop treated you differently because of your role in the program?*
