# Supplementary material for: Cutting Out Stigma: Findings From a Pilot Implementation Trial of a Barbershop‐Based HIV Stigma Reduction and Health Promotion Intervention in the Southern United States
Source: J Int AIDS Soc. 2026 Oct 8;29(Suppl 5):e70195. doi: 10.1002/jia2.70195 (PMC13650421; doi:10.1002/jia2.70195)
Supplement: Supplementary file 2 [file JIA2-29-e70195-s002.docx]

Supplemental Table 1. Integration of quantitative effectiveness results and qualitative findings from Cutting Out Stigma, with barber-reported early client indicators.

| **Effectiveness domain (barber-level)** | **Quantitative result** | **Qualitative finding** | **Integration: fit, meta-inference, and client indicators** |
| --- | --- | --- | --- |
| **Perceived effectiveness in the COS role (incl. shop-level stigma reduction)** | **Self-reported effectiveness as Men’s Health Ambassador:**  median 8 (IQR 7 to 9) | Barbers adopted a trusted community-leader role, using nonjudgmental conversation to reduce shop-level stigma, supported by COS positive-framing visual and social-media cues.  Contact with people living with HIV (through training or personal ties) reduced barbers’ own fear and judgment; about half navigated “judgment by association” when raising HIV, then honed nonjudgmental responses.  HIV talk became normalized as “shop talk,” with some reporting less stigmatizing language; privacy and gender norms still constrained openness. Influence extended beyond the shop, including parents inviting barbers to speak with their children.  *“Not judging… helped [clients] open up… cutting out the stigma.” (Barber A)* | **Confirmation + expansion.** The score confirms role effectiveness; the qualitative data locate its mechanism in stigma reduction (via knowledge and contact with PLWH) and show the role spreading into the wider community, while privacy and gender norms temper it.  **Client (barber-reported):** clients more comfortable and open discussing HIV and sexual health; conversations spilling beyond the shop. |
| **Change in barbers’ HIV-related knowledge** | **HIV knowledge (0 to 21 scale):**  rose from 15.0 to 16.9 after training; p < 0.01 (n = 50) | HIV reframed from fatal and exceptional to manageable and preventable; training and ongoing coaching built familiarity with PrEP and U=U (undetectable equals untransmittable).  Certified expertise and accurate information gave barbers credibility with clients and underpinned stigma reduction by dismantling fatal-disease myths.  *“I thought it was a terminal death sentence… there’s a pill you can take regularly… you can live a good, full, healthy… life.” (Barber A)* | **Confirmation + expansion.** The pre to post gain confirms the change; the qualitative data show its de-stigmatizing character and its role in building barber credibility.  **Client (barber-reported):** clients, especially younger ones, gained accurate HIV and PrEP information through trusted barber conversations. |
| **Referrals to community resources** | **Weekly referrals (per barber):**  0-5: 57%; 6-10: 25%; 11+: 18%  **Top services:**  HIV testing 71%; PrEP 70%; primary care 49% | Talk-to-action beginnings: condom uptake, testing requests, PrEP inquiries, and some barbers modeling testing themselves. Visual reminders of COS resources and prevention kits supported movement from misinformation to action.  Intention-behavior gaps persisted, driven by anticipated stigma, lack of privacy, gender norms, and client perceived invincibility.  *“Client… was willing to go to a local clinic with me… interested in PrEP.” (Barber H)* | **Confirmation with discordance.** Referral counts and the testing/PrEP-weighted mix confirm the conversation-to-action pathway; referral distribution converges with the barber-described intention-behavior gap for some clients.  **Client (barber-reported):** observed condom uptake, testing and PrEP inquiries, and willingness to be referred to services. |

*Notes:* Domains are the a priori RE-AIM Effectiveness sub-domains (upstream barber effects). Community stigma reduction is treated as cross-cutting, centered in the COS role and woven through the knowledge and referral domains; Fit is from Fetters et al., 2013: confirmation, qualitative accounts corroborate the quantitative result; expansion, they elaborate its mechanisms; discordance, they diverge from it

Abbreviations: COS, Cutting Out Stigma; PrEP, pre-exposure prophylaxis; U=U, undetectable equals untransmittable; PLWH, people living with HIV; IQR, interquartile range.
